# Phenolic Composition, Antioxidant Capacity and Antibacterial Activity of White Wormwood (*Artemisia herba-alba*)

**DOI:** 10.3390/plants10010164

**Published:** 2021-01-16

**Authors:** Muthanna J. Mohammed, Uttpal Anand, Ammar B. Altemimi, Vijay Tripathi, Yigong Guo, Anubhav Pratap-Singh

**Affiliations:** 1 Department of Biology, College of Education for Pure Sciences, University of Mosul, Mosul 41002, Iraq; dr.muthanna.j.m@uomosul.edu.iq; 2 Department of Molecular and Cellular Engineering, Jacob Institute of Biotechnology and Bioengineering, Sam Higginbottom University of Agriculture, Technology and Sciences, Prayagraj 211007, India; ushuats@gmail.com (U.A.); vijay.tripathi@shiats.edu.in (V.T.); 3 Department of Food Science, College of Agriculture, University of Basrah, Basrah 61004, Iraq; 4 Food, Nutrition and Health Program, Faculty of Land and Food Systems, The University of British Columbia, Vancouver, BC V6T 1Z4, Canada; yigong.guo@ubc.ca

**Keywords:** *Artemisia herba-alba*, extracts, phenolics, antimicrobial effects, antioxidant effects

## Abstract

*Artemisia herba-alba* Asso. (Wormwood) is a wild aromatic herb that is popular for its healing and medicinal effects and has been used in conventional as well as modern medicine. This research aimed at the extraction, identification, and quantification of phenolic compounds in the aerial parts of wormwood using Soxhlet extraction, as well as characterizing their antimicrobial and anitoxidant effects. The phenolic compounds were identified in different extracts by column chromatography, thin layer chromatography (TLC), and high performance liquid chromatography. Five different fractions, two from ethyl acetate extraction and three from ethanolic extraction were obtained and evaluated further. The antimicrobial activity of each fractions was evaluated against two Gram-positive (*Bacillus cereus* and *Staphylococcus aureus*) and two Gram-negative microorganisms (*Escherichia coli* and *Proteus vulgaris)* using the disc-diffusion assay and direct TLC bioautography assay. Fraction I inhibited *B. cereus* and *P. vulgaris*, Fraction II inhibited *B. cereus* and *E. coli*, Fraction III inhibited all, except for *P. vulgaris*, while Fractions IV and V did not exhibit strong antimicrobial effects. Their antioxidant capabilities were also measured by calculating their ability to scavenge the free radical using DPPH method and the ferric reducing antioxidant power (FRAP) assay. Ethanolic fractions III and V demonstrated excellent antioxidant properties with IC_50_ values less than 15.0 μg/mL, while other fractions also had IC_50_ values less than 80.0 μg/mL. These antioxidant effects were highly associated with the number of phenolic hydroxyl group on the phenolics they contained. These extracts demonstrated antimicrobial effects, suggesting the different phenolic compounds in these extracts had specific inhibitory effects on the growth of each bacteria. The results of this study suggested that the *A. herba-alba* can be a source of phenolic compounds with natural antimicrobial and antioxidant properties which can be used for potential pharmaceutical applications.

## 1. Introduction

In recent years, traditional plants have emerged as potential sources of antioxidants, antimicrobials, and secondary metabolites for therapeutic interventions, which has opened doors for the development of novel plant-based antibacterial agents [1,2,3,4,5]. At the same time, evolving consumer outlook has created increased importance for the nutritive value of the food [6], skyrocketing the need for finding novel sources of healthy phenols and antioxidants. *Artemisia herba-alba* Asso. is widely known as desert wormwood, that is extensively used in conventional and herbal medicine for the treatment of diabetes, parasitic infection, hypertension and cold [6,7]. *A. Herba-alba* mainly belongs to the genus *Artemisia* which can generally grow in the semi-arid region of the Mediterranean (Middle East), North Africa, Spain, and the northwest region of the Himalayas [8]. The *Artemisia* genus has approximately 400 species which are mostly diploid and tetraploid. *Artemisia herba-alba* plant is a green perennial shrub that grows 20–50 cm long with small and hairy leaves [9]. Flowering starts from September to December but the full development begins at the end of the summer with woolly hairs stem [10,11]. From ancient times, the plant extract of *A. Herba-alba* has been used as a traditional medicine in many cultures because it has many pharmacological and biological activities especially antidiabetic, antimicrobial, antitumor, antimalarial, antioxidant, insecticidal and neurological activities [12,13,14,15,16,17,18]. In Morocco, this plant has been used in herbal tea to treat hypertension and stomach disorder [19]. In Algeria, this plant is used as fodder for lamb and other livestock [20,21,22].

In recent decades, many researchers have conducted phytochemical analysis of *A. Herba-alba* in several countries [23,24,25]. The terpenoid sesquiterpene lactone dehydroleucodine, mainly found in the aerial parts of *A. herba-alba*, is responsible for its medicinal properties [26]. Various volatile compounds, such as chrysanthenyl acetate, chrysanthenol, acetophenone xanthocyclin, 1,8-cineole, α- and β-thujone, terpinen-4-ol, camphor, and borneol, were observed in different collected populations of *A. Herba-alba* plants in Eilat and Judian desert region of Israel [27] and also in Morocco [28]. The monoterpenes and α- and β-thujones were dominant in Jordanian populations but sabinyl acetate, germacrene D, α-eudesmol and caryophyllene acetate were also identified [27]. Essential oils of *Artemisia* genus plants were reported to have antibacterial activity against some pathogenic bacteria [29]. Very recent investigations from Egypt revealed two new antimicrobial compounds (metabolites), namely 1,3,8-trihydroxyeudesm-4-en-7α,11βH-12,6α-olide and 5-β-D-glucopyranosyloxy-7-methoxy-6H-benzopyran-2-one from the aerial parts of *A. herba-alba* [30]. Aforementioned researches evaluated basic phytochemical profile of the *A. herba-alba* extracts, however, individual compounds responsible for the medicinal effects have not been extracted, and their characteristics are still to be documented. 

Thus, the aim of this study was to extract the fractions of different phenolic compounds of *Artemisia herba- alba* (Wormwood) growing in Iraq by chromatographic methods, namely column chromatography (CC), thin layer chromatography (TLC), and high performance liquid chromatography (HPLC). The antimicrobial activity was evaluated against bacterial pathogens using the disc diffusion assay and TLC bioautography assay for checking the inhibitory effect of extracted phenolic compounds. The novelty of this study is that it describes for the first time use of Soxhlet apparatus for the extraction of phenolic compounds from *A. herba-alba* plant.

## 2. Results and Discussion

### 2.1. Composition of Phenolic Compounds in A. herba-alba Fractions

**Figure 1:**
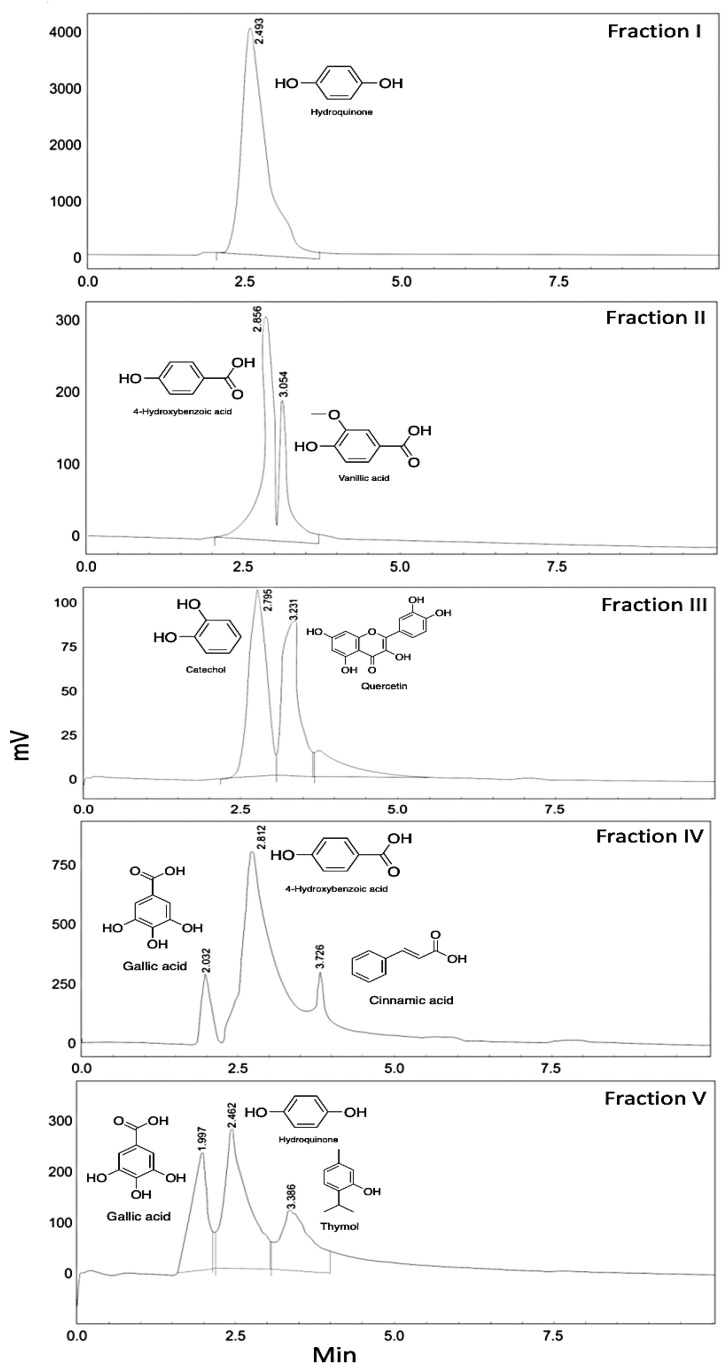
HPLC chromatogram of Fraction I-IV.

One of the most abundant bioactive components of *Artemisia herba-alba* are phenolic compounds [31]. Five fractions were collected after extraction. The fractions I and II were related to ethyl acetate extraction, while the fractions III, IV and V were related to ethanol extraction. Table 1 shows the composition and content of phenolic compounds in the different fraction parts of *A. herba-alba* determined by HPLC-analysis under 280 nm. The composition and content of phenolic compounds were identified by comparing their retention times and peak areas with each standard (Section 3.5). In Fraction I, one major peak was obtained in the HPLC chromatographic profile (Figure 1), which were identified as hydroquinone on the basis of its standard. In Fraction II, two major peaks were obtained, which were identified as 4-hydroxy benzoic acid and vanillic acid (Figure 1). Moreover, two phenolic compounds, catechol and quercetin, were obtained in Fraction III (Figure 1). Three phenolic compounds, gallic acid, 4-hydroxy benzoic acid and cinnamic acid, were found in Fraction IV, while gallic acid, hydroquinone, and thymol were identified in Fraction V (Figure 1). The remaining phenolic components were present in very trace amounts that could not be extracted or quantified by this method. Based on the result shown in Table 1, the major phenolic compounds in the extract were hydroquinone, 4-hydroxy benzoic, cinnamic acid and thymol. Younsi et al. [23] found a total phenolic composition of 27.65 mg GAE/g dry weight, while our results demonstrated about 88 mg/g dry weight of phenolics. Compared with previous studies [32], the content of cinnamic acid could be up to 10.52%, which was consistent with the results of this study. Interesting, thymol, hydroquinone and 4-hydroxy benzoic acid were found at low contents in *A. herba-alba* previously [25,33,34], which were different from the results of this study. The reason for higher yield in our study, was probably due to the use of Soxhlet apparatus, which can result in high yield of compounds with low solubility. Moreover, low contents of vanillic acid, catechol, quercetin, and gallic acid were also found in the extracts. These phenolics exert antioxidant properties, which could be used to regulate the kinetics of various degradation reactions affecting food quality [35]. The components herein reported in the *A. herba-alba* were similar to those reported in previous studies of these compounds in the aerial parts of *A. herba-alba* [23,25,36], with varying contents on account of the extraction method and origin of plant materials. 

### 2.2. Antimicrobial Activities of A. herba-alba Extracts

The antibacterial activity of the combined *A. herba-alba* extracts were tested by direct TLC bioautography assay. Table 2 describes the retardation factors (R*_f_*) of the various inhibition zones recorded on the plate. In general, six different inhibition zones were recorded at R*_f_* = 0.20, 0.30, 0.40, 0.55, 0.70, and 0.80. For gram positive bacteria, *S. aureus* and *B. cereus* recorded three (R*_f_* = 0.20, 0.40, 0.70) and five (0.30, 0.40, 0.55, 0.70, 0.80) inhibition zones were recorded respectively. For Gram-negative bacteria, *E. coli* and *P. vulgaris* showed three (0.40, 0.55, 0.70) and one (R*_f_* = 0.30) inhibition zones respectively. The antibacterial activity of different fractions of *A. herba-alba* extracts were also tested by disc diffusion method against same 4 bacterial strains. Based on the results shown in Table 2, these five extracts exhibited antibacterial activities against most bacteria. *S. aureus* were inhibited at concentrations of 1.25 μL/mL for Fraction III, 2.5 μL/mL for Fraction IV and V and 5 μL/mL for Fraction I and II. *B. cereus* were inhibited at concentrations of 1.25 μL/mL for Fraction I, II and III, 2.5 μL/mL for Fraction IV and 5 μL/mL for Fraction V. *E. coli* were inhibited at concentrations of 1.25 μL/mL for Fraction II and III, 2.5 μL/mL for Fraction IV and V and 10 μL/mL for Fraction I. *P. vulgaris* were inhibited at concentrations of 1.25 μL/mL for Fraction I, 5 μL/mL for Fraction II, 10 μL/mL for Fraction IV and V and 20 μL/mL for Fraction III. Fraction I could mostly inhibit the growth of *B. cereus* and *P. vulgaris* and showed low inhibition effects on *S. aureus* and *E. coli*. This is consistent with the fact that the major compound in this Fraction is hydroquinone, which can inhibit the growth of *B. cereus* and *P. vulgaris* efficiently [37]. This is also consistent with the R*_f_* = 0.30 inhibition zone visible for B. cereus and P. vulgaris strains on the TLC-bioautography assay. For Fraction II, the inhibition zone of *B. cereus* and *E. coli* were more significant than the other two strains, which was also depicted by the inhibition zones at R*_f_* = 0.40 and 0.55 for *B. cereus* and *E. coli* in the TLC bioautography assay. The reason for the stronger inhibition effects of this Fraction is the presence of the vanillic acid. Previous study showed that it exhibited high antimicrobial effect toward *B. cereus* and *E. coli* [38]. In this study, due to the occurrence of quercetin, Fraction III showed the broad antimicrobial effect as it inhibited almost all the strains used in this test except for *P. vulgaris*. This is depicted in the TLC bioautography assay by R*_f_* = 0.40 and 0.70 in all microbes except *P. vulgaris*. Based on previous study, quercetin can potentially inhibit the growth of *S. aureus*, *B. cereus* and *E. coli* [39]. However, Fraction IV and Fraction V did not show strong inhibition effects on all the strains used in low concentration. When the concentration of these two fractions increased, they also showed potential antimicrobial effects. That is probably because gallic acid was found in both of these fractions, which has been proven to have moderate antimicrobial activities [40]. Noticeably, *P. vulgaris* is resistant to most of the extracts in this study, except Fraction III. This could be related to its outer membrane nature, which gives it intrinsic resistance to antimicrobials and antibiotics [41]. Interestingly, this is the first study focussed on different extraction fractions of *A. herba-alba* using Soxhlet apparatus. On the other hand, it should be emphasized it is possible that some compounds in the extract could give rise to the antibacterial effects by synergistic interactions with other compounds, as most of them contained more than one phenolic compound. Moreover, the disc diffusion assay itself is a test mainly based on the diffusibility and solubility of the sample added. Thus, it must be mentioned that the individual compounds in these fractions must be tested separately before attributing the observed antimicrobial action to any individual compound.

### 2.3. Antioxidant Activities of A. herba-alba Extraction

**Figure 2:**
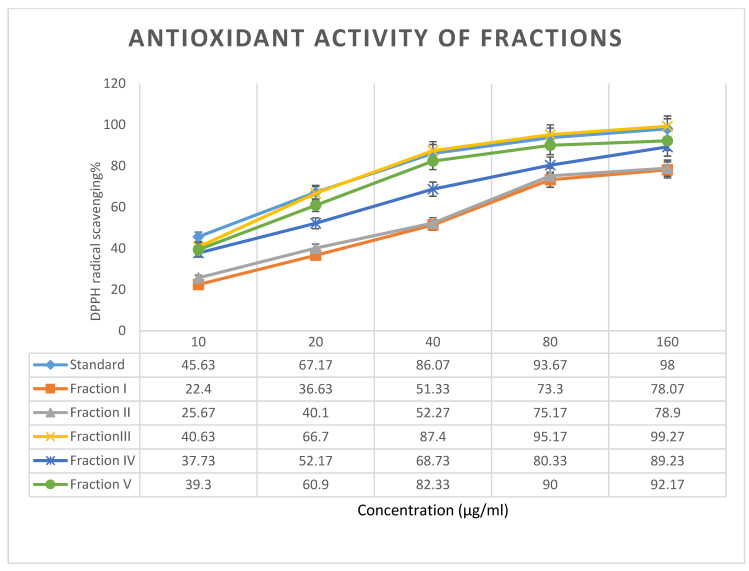
Antioxidant and free radical scavenging activity of isolated fractions.

The DPPH free radical method was used to measure the antiradical power of *A. herba-alba* extracts in a recent study [42]. Figure 2 showed the abilities of different fractions of *A. herba-alba* to reduce DPPH radicals. Compared with the standard, Fraction III showed similar antioxidant effect, followed by Fractions V and IV, while Fractions I and II exhibited relatively weak antioxidant effects compared with other fractions. All fractions in this study showed antioxidant effects, although the antioxidant strength varied as different phenolics compounds have different redox properties, which make them reducing agents, hydrogen donors, and singlet oxygen quenchers. Since one major component of Fraction III was quercetin, which was also used as standard in this study, it had very similar antioxidant effect as the standard (Figure 2 and Table 3). In addition, based on previous research, the efficiency of phenolic compounds as anti-radicals and antioxidants depends on many factors. One major factor is the number of hydroxyl groups directly bonded to the aromatic rings [43]. This structure is related to the stability of the hydroxyl radical formed after phenolics donate their hydrogen atoms to the radical. In this study, gallic acid was found in both Fractions IV and V. The three phenolic hydroxyl group of gallic acid made it the second strongest antioxidant among all the phenolic compounds identified in this study. As a result, Fractions IV and V also showed relatively strong antioxidant effects in this study. In contrast, all phenolic compounds found in Fractions I and II only had two or less hydroxyl groups connected to their aromatic rings, which led to the low antioxidant effects of these two fractions. However, based on previous study [44], different solvent used in plant extract would also impact the DPPH results, which is consistent with the result shown in this research. From the DPPH results, the IC_50_ values were obtained as 12.0 μg/mL for standard, 38.2 μg/mL for Fraction I, 36.3 μg/mL for Fraction II, 13.6 μg/mL for Fraction III, 18.5 μg/mL for Fraction IV and 15.0 μg/mL for Fraction V. Further, Younsi et al. [23] reported an IC_50_ value of 100 μg/mL for methanolic *A. herba-alba* extracts, whereas the antioxidant effects of our extracts were higher demonstrating the superiority of the Soxhlet apparatus-based extraction method employed in our study. It must also be mentioned that DPPH inherently is a limited test on antioxidant activity, and at least one more method for measuring antioxidant efficacy is necessary. 

Another antioxidant activity test used in this study was ferric reducing antioxidant power (FRAP) assay. According to previous study [45], FRAP assay has been used to evaluate antioxidant activity of various compounds because it is reproducible and linear related to molar concentration of the antioxidants inside the testing samples. Since antioxidant compounds in *A. herba-alba* extracts can produce a color complex with potassium ferricyanide, trichloro acetic acid, and ferric chloride, the increase in absorbance under 700 nm of the reaction mixture indicates the possibility of using these extracts as potential antioxidants [46]. The results (Table 3) showed that the fraction III had the strongest ferric reducing antioxidant power followed by Fractions V and IV, while Fractions I and II exhibited relatively weak ferric reducing antioxidant effects compared with other fractions. Noticeably, fraction III even exhibited higher effect than the standard in high concentration (>40 μg/mL). FRAP IC_50_ were reported as 9.8 μg/mL for standard, 40.4 μg/mL for Fraction I, 76.9 μg/mL for Fraction II, 8.3 μg/mL for Fraction III, 20.2 μg/mL for Fraction IV and 13.8 μg/mL for Fraction V. Sendi et al. [47] reported IC_50_ of 10 μg/mL for antioxidant properties of A. *herba-alba* extracts after optimization of extraction conditions. Our values were close to that value, without optimization, and further optimization of extraction might result in even higher antioxidant activity, confirming the superiority of the Soxhlet extraction procedure applied in this study. All the results in this assay were consistent with the results got from DPPH assay. High antioxidant capacity depicted by FRAP value suggests that antioxidants in *A. herba-alba* extracts were capable of donating a single electron or hydrogen atom for reduction. It has been mentioned that FRAP assay creates problems with some antioxidants such as gluthathione [48], as the speed of reaction is not fast enough. However, such compounds were not detected in *A. herba-alba* extracts and thus the FRAP assay can still be used for assessment of antioxidant activity in *A. herba-alba* extracts.

## 3. Materials and Methods

### 3.1. Materials

*Artemisia heba-alba* Asso.’s aerial (without pathogenic and physical damage) parts were collected in April and June 2019 from northern Iraq during the flowering season, when temperatures typically vary from a night-time low of 20 °C to a day-time high of 38 °C. The cultivated plants were grown at Mosul (20′ 24.0000″ N and 43° 7′ 48.0036″ E), which is a major city in northern Iraq. The soil used during cultivation of the plant primarily comprised of silt and sand. The choice for the geographical location was based on expert suggestion and literature survey. The plant parts were collected, wearing gloves to maintain sterility, and were subsequently kept in sterile plastic bag before being transported to the laboratory. Collected plant materials were identified/authenticated by personnel from Ministry of Agriculture, Baghdad, Iraq. After confirmation of plant identity, it was systematically washed with sterile distilled water to remove all surface dust particles. After this, plant materials were immediately placed in dark at room temperature (RT, approximately 25 °C) until dry, as phenolics are prone to photodegradation [49]. Once the plant material was dried at RT, it was subsequently transferred into a hot air oven (HAO) for drying at 36 °C for around 48 h. Eventually, the dried plant parts were vigorously crushed into a very fine powder/paste using a pre-chilled laboratory mortar and pestle. Following crushing, the plant powder was filtered through sterile muslin cloth and subsequently safely stored at −20 °C for further investigation. All reagents (hexane, ethyl acetate, ethanol, methanol, chloroform, vanillin sulphuric acid, acetonitrile, phosphoric acid, hydroquinone, 4-Hydroxy benzoic acid, vanillic acid, catechol, quercetin, gallic acid, cinnamic acid, and thymol) used for extraction, isolation, and analysis were of analytical grade and obtained from Sigma Aldrich (Baghdad, Iraq).

### 3.2. Extraction of Artemisia by Soxhlet Apparatus

Firstly, 5 g of crushed plant powder/paste was processed/extracted using Soxhlet apparatus successively with different conventional organic solvents (100 mL), namely hexane, ethyl acetate and ethanol, in order of increasing polarity. The extraction process was carried out at constant temperature of 60 °C for 72 h at water bath, and was repeated thrice for each solvent. A rotary evaporator was used to evaporate the crude plant extract. Once the organic solvent evaporated, the extract was filtered with a 0.22 μm polytetrafluoroethylene (PTFE) filter to remove undesired materials. All extracts were weighed and stored in sterile dark airtight containers for further analysis, as phenolics are prone to photodegradation [49].

### 3.3. Isolation and Fractions of Artemisia Extract by Column

Column chromatography (CC) was performed for isolation and fractionation of *Artemisia* extract. Chromatography column was packaged using silica gel (300 g, Sigma Aldrich, Baghdad, Iraq) (60–120) as an adsorbent using wet packaging technique. Using a colourless liquid (hexane), slurry was prepared and poured into the column. After mixing them homogeneously with a small amount of silica gel over the top of the column, the extract was added. For column elution systems, different solvent mixtures were used as mobile phase including hexane, ethyl acetate and ethanol. For the identification of fractions that contain *Artemisia*, collected fractions following purification of the CC were finally examined with thin layer chromatography (TLC). Collected fractions were condensed and only ethyl acetate and ethanol fractions were subsequently identified by TLC because the hexane fraction was very hard to separate and came up as crude sample.

### 3.4. Thin-Layer Chromatography (TLC)

To select an appropriate solvent system, for separating the various components present in the crude extract, TLC of the crude extract was performed using a method detailed earlier [32]. For detecting the presence of phytochemicals, an aluminium-backed thin layer chromatography (TLC) apparatus was loaded with 2 μL of each extract. The glass TLC plates were 20 cm by 20 cm and pre-coated with silica gel 60 F254 (E. Merck/Millipore, Billerica, MA, USA, 0.2 mm thickness). A solution of chloroform, ethyl acetate and formic acid, in a ratio of 10:8:2 *v/v* was used to develop the TLC plate. The developed plate was then viewed at 254 nm and 365 nm under ultra-violet light for fluorescent compounds using a UV-visible spectrophotometer (Sunny UV.7804C, Tokyo, Japan). Next, the TLC plate was sprayed with vanillin sulphuric acid and subsequently heated to visualize colors of the different compounds. The R*_f_* value was determined for each position of the detected spot and compared with standards. Fractions with same R*_f_* values were separated and each of them were concentrated using rotary evaporator. When dried, weight measurements were taken, and the condensed fractions were further analyzed using HPLC (High-performance liquid chromatography) technique for confirmation of the identity of phenolic compounds.

### 3.5. Analysis of Phenols by HPLC (High Performance Liquid Chromatography)

HPLC was employed to confirm the identity of phenolic components extracted. With its high sensitivity and speed, HPLC represent one of the best analytical system to analyze plant substances such as phenols. The analytical HPLC system (reversed phase HPLC with silica-based C18; Agilent Technologies, Santa Clara, CA, USA) consisted of a detector for SPD-10A UV-VIS, VP pump LC-10AT, an auto injector SIL-10AF, and the system controller SCL-10A VP. The analytical column was the Chiralcel ^®^ OD-RH (150 mm roughly 4.6 mm diameter, 5 mm particle size Chiral Technologies Inc. Exton, PA, USA). The mobile phase used was acetonitrile, water and phosphoric acid (30:70:0.08, *v/v/v*) under isocratic conditions at ambient temperature (25 ± 1 °C) with a flow rate of 0.4 mL/min. Each run was 8 min, followed by 15 min for clean-up. Separated compounds were detected by the built-in SPD-10A UV-Vis detector at 288 nm. Overall, the phenolic compounds were identified by the method described by Skendi et al. [50]. The identity of individual compounds was confirmed by matching the retention time of respective standards (Table 4) with those of the peaks in the extract.

### 3.6. Origin and Selection of Microbial Strains

The *Artemisia herba-alba* extracts were examined against a series of highly pathogenic microorganisms. In this study, four human pathogenic bacteria were selected to screen their in-vitro antimicrobial activities. All four microbial strains were obtained from the stock culture of our laboratory. Both Gram-positive strains, including *Bacillus cereus* (ATCC 14579), *Staphylococcus aureus* (ATCC 6538), and Gram-negative strains, including *Escherichia coli* (ATCC 8739) and *Proteus vulgaris* (ATCC 7829), were tested in this study, as these are the most common food borne pathogens of concern. Bead vials were used to safely store microorganisms in frozen condition (−18 °C).

### 3.7. Preparation of the Inoculums

The bacterial strains were grown/cultured onto the nutrient agar (NA) favourable for their growth (Mueller-Hinton broth) at 37 °C for 24 h. Following incubation, they were sub-cultured before any antimicrobial test. To prepare inoculums, bacteria were suspended in a sterile saline solution (0.85% NaCl). The optical density (OD) of the suspensions were adjusted/maintained from 0.4 to 0.6 at 405 nm, which corresponds to a cell density close to that of 0.5 McFarland, matching to an inoculum estimated at 10^6^ to 10^8^ colony forming units per mL (CFU/ mL) [51].

### 3.8. Disk Diffusion Method on Agar

The antibiotic susceptibility test was carried out by the conventional disk diffusion technique as explained by Zazharskyi et al. [52] with some modifications. In this method, the Mueller-Hinton agar (MHA) plates were streaked by previously prepared inoculums using a sterile swab. Then, 5 μL of each ethyl acetate (I-II) and ethanol (III-V) fractions were impregnated onto sterilized paper discs (6 mm, Whatman paper N°5) in solvent (10% *v/v* dimethyl sulfoxide and 1% *v/v* tween 80 in deionized water). Under same conditions, the antibiotics Amikacin and Gentamycin (5 μg/mL) and the same solvent (10% *v/v* dimethyl sulfoxide and 1% *v/v* tween 80 in deionized water) employed in the dilution of extract fractions were used as positive and negative control respectively. The plates were maintained at a room temperature (RT) and then incubated for 24 h at 37 °C. In the end, antibacterial activity was evaluated by measuring in millimetres the nearest surrounding diameter of resulting inhibition zones (around and including discs diameter). Each experiment was done in triplicates.

### 3.9. Direct TLC Bioautography Assay

The developed TLC plates from Section 3.4. were used for Direct bio-autography of the fractions of *Artemisia herba-alba* against same four microbial strains identified in Section 3.6 (*S. aureus*, *B. cereus*, *E. coli*, and *P. vulgaris*) using a method described by Khaleel et al. [53]. Plates were dried overnight (to evaporate excess solvents) and then sterilized under UV light for 15 min under laminar flow. 100 μL standardized bacterial suspension was pipetted onto the plate surface and spread evenly with an L-shaped glass rod, followed by a careful placement of the TLC plates inside a square plastic box and incubation at 30 °C for 24 h. Next, the plates were inoculated with bacterial suspension sprayed with 5% 2,3,5-Triphenyl-2H-tetrazolium chloride (TTC) aqueous solution that stained the TLC plate background with viable bacteria cell when left for 4 h under laminar flow. The inhibition zones, detected as a white background, was represented by individual retardation factors (R*_f_*) values, determined as the ratio of the distance moved by the compound from its origin to the movement of the solvent from the origin.

### 3.10. DPPH Radical Scavenging Activity Assay

DPPH (2,2-diphenyl 1-picryl hydrazyl), a well-established organic chemical for the analysis of free radical scavenging capacity was used as previously described by Amiri et al. [54] with slight modifications. The radical scavenging activity was carried out using free-radical DPPH assay using the spectrophotometric approach. The fractions are prepared in different concentrations (10, 20, 40, 80, 160) μg/mL and then dissolved in 1 mL of ethanol and 20 mg of DPPH dissolved in 100 mL ethanol was added. The mixture was shaken, and then left for 30 min at RT under dark conditions. For control, DPPH solution was taken, whereas for reference standard ascorbic acid (water-soluble vitamin) was employed. The antioxidant activity was then calculated using the UV-Visible spectrophotometer at 517 nm. Radical scavenging activity was calculated by the following relationship:

DPPH Inhibition % = [(A_o_ − A_1_)/A_o_] × 100

where A_o_ is the control test absorbance after 30 min and A_1_ is the sample extract absorption after 30 min.

### 3.11. Ferric Reducing Antioxidant Power (FRAP) Assay

The antioxidant capacity was also evaluated using ferric reducing antioxidant power (FRAP) assay. In order to determine the ferric reducing activity of different fraction of *A. herba-alba* extracts, FRAP assay was performed based on the methods of Oyaizu [55] with slight modification. Briefly, all fractions in different concentrations (10, 20, 40, 80, 160 μg/mL) and standard (ascorbic acid) were mixed with 1 mL of distilled water, 2.5 mL of K_3_Fe(CN)_6_ (1% *w/v*) and 2.5 mL of 0.2 M phosphate buffer (pH 6.6). 22.5 mL of trichloro acetic acid (10% *w/v*) were then added in to these mixtures after a 20 min incubation at a temperature of 50 °C. All mixtures were centrifuged at 3000 rpm for 10 min. The supernatant (2.5 mL) of each testing samples were mixed with 2.5 mL of distilled water and 0.5 mL of FeCl_3_ (0.1%, *w/v*). Finally, the ferric reducing antioxidant power was calculated according to the absorbance at 700 nm using a spectrophotometer (Sunny UV.7804C, Tokyo, Japan) by following equation:

% ferric reducing antioxidant power = (A/A_0_) × 100%

where A is the absorbance of sample and A_0_ is the absorbance of control (deionized distilled water).

### 3.12. Statistical Analysis

All experiments were performed in triplicates (*n* = 3), and datasets obtained were analyzed by different statistical methods. The statistical analysis was carried out using the Statistical Package for the Social Sciences (SPSS) program using analysis of variance (ANOVA) to investigate the effect of *Artemisia* extract fractions. Mean comparison using Tukey’s test was performed with STATISTICA 13 (alpha = 0.05). Microsoft Excel (2007) and BioStat were used to prepare the graphs and results.

## 4. Conclusions

The results obtained in this study showed the composition, antimicrobial and antioxidant properties of different *A. herba-alba* Asso. extracts. Eight phenolic compounds were identified. This study demonstrated for first time the use of Soxhlet apparatus for extraction of *A. herba-alba* aerial parts, which resulted in higher yield of phenolics such as thymol, hydroquinone and 4-hydroxy benzoic acid, as compared to previous reports. Fractions of *A. herba-alba* extracts depicted different levels of antimicrobial activities against the bacteria assayed in this study. Fraction I could efficiently inhibit the growth of *B. cereus* and *P. vulgaris*, Fraction II showed strongest inhibition effect toward *B. cereus* and *E. coli*, Fraction III showed the broad antimicrobial effects again all microorganisms tested, except for *P. vulgaris*, while Fractions IV and V did not exhibit strong antimicrobial effects in low concentrations. In terms of antioxidant effects, Fraction III showed strongest antioxidant effect, followed by Fractions V and IV, with Fractions I and II being the weakest. This difference was attributed to the phenolic compounds identified in each fraction, with the intensity of antioxidant effects strongly correlated to the number of hydroxyl groups. Further in vivo studies and clinical assays are still needed to confirm the safety and possible applications of these *A. herba-alba* extracts as antimicrobial and antioxidant agents. Results from this study could be used to further develop a powerful method for extraction of phenolic compounds from white wormwood wood for use in various nutraceutical and pharmaceutical applications.

## Figures and Tables

**Table 1:** Phenolic compounds in different fractions and their retention time.

Fractions	Number of Peak	Retention Time (min)	Concentration (ppm) ^c^	Identifed Compounds
**I ^a^**	1	2.5	18.0 ± 0.9	Hydroquinone
**II ^a^**	1	2.9	2.3 ± 0.2	4-Hydroxy benzoic acid
	2	3.1	2.2 ± 0.3	Vanillic acid
**III ^b^**	1	2.8	0.7 ± 0.1	Catechol
	2	3.2	1.6 ± 0.2	Quercetin
**IV ^b^**	1	2.0	5.1 ± 0.3	Gallic acid
	2	2.8	14.1 ± 0.7	4-Hydroxy benzoic acid
	3	3.7	20.3 ± 1.1	Cinnamic acid
**V ^b^**	1	2.0	4.6 ± 0.3	Gallic acid
	2	2.5	1.8 ± 0.2	Hydroquinone
	3	3.4	14.9 ± 0.8	Thymol

^a^ Fractions identified from ethyl acetate extraction; ^b^ Fractions identified from ethanol extraction; ^c^ Values represent mean and standard deviation (*n* = 3).

**Table 2:** Antimicrobial activity of fraction I–V.

	Concentration	Zone of Inhibition (mm) ^e^			
Fraction I (2)	**μg/mL**	***S. aureus***	***B. cereus***	***E. coli***	***P. vulgaris***
	1.25	0	8 ± 1.12 ^a^	0	9 ± 1.02 ^a^
	2.5	0	15 ± 0.87 ^b^	0	12 ± 0.95 ^a^
	5	10 ± 1.01 ^a^	17 ± 1.02 ^b^	0	15 ± 1.03 ^b^
	10	13 ± 1.11 ^a^	19 ± 0.99 ^c^	12 ± 0.87 ^a^	18 ± 1.11 ^c^
	20	17 ± 0.88 ^b^	20 ± 1.07 ^c^	17 ± 1.03 ^b^	19 ± 0.99 ^c^
Fraction II (3,4)	1.25	0	11 ± 1.02 ^a^	10 ± 1.1 ^a^	0
	2.5	0	16 ± 1.02 ^b^	12 ± 1.09 ^a^	0
	5	11 ± 0.91 ^a^	18 ± 0.82 ^b^	17 ± 0.84 ^b^	12 ± 0.88 ^a^
	10	16 ± 1.04 ^b^	21 ± 1.03 ^c^	21 ± 1.07 ^c^	16 ± 1.01 ^b^
	20	17 ± 1.12 ^b^	22 ± 0.97 ^c^	20 ± 0.99 ^c^	22 ± 0.95 ^c^
Fraction III (3,5)	1.25	10 ± 0.97 ^a^	12 ± 1.04 ^a^	8 ± 0.98 ^a^	0
	2.5	13 ± 1.03 ^b^	16 ± 0.92 ^b^	12 ± 1.03 ^a^	0
	5	18 ± 1.01 ^c^	20 ± 0.89 ^c^	15 ± 1.04 ^b^	0
	10	19 ± 0.98 ^c^	25±0.93^d^	17 ± 0.99 ^b^	0
	20	20 ± 0.92 ^c^	27 ± 1.05 ^d^	20 ± 1.09 ^c^	9 ± 1.05 ^a^
Fraction IV (1,3,6)	1.25	0	0	0	0
	2.5	13 ± 1.11 ^a^	8 ± 1.02 ^a^	10 ± 1.05 ^a^	0
	5	16 ± 0.91 ^b^	14 ± 0.98 ^b^	17 ± 0.87 ^b^	0
	10	19 ± 1.08 ^c^	18 ± 1.03 ^c^	17 ± 1.08 ^b^	14 ± 0.88 ^b^
	20	25 ± 1.01 ^d^	20 ± 0.91 ^c^	19 ± 1.01 ^b^	18 ± 1.03 ^c^
Fraction V (1,2,5)	1.25	0	0	0	0
	2.5	11 ± 1.2 ^a^	0	15 ± 0.83 ^b^	0
	5	16 ± 1.3 ^b^	15 ± 1.12 ^b^	17 ± 1.04 ^b^	0
	10	23 ± 0.98 ^c^	17 ± 0.93 ^b^	21 ± 1.12 ^c^	11 ± 0.94 ^a^
	20	21 ± 1.11 ^c^	20 ± 1.11 ^c^	21 ± 1.14 ^c^	17 ± 1.02 ^b^
Control	Amikacin	22	24	25	23
	Gentamycin	25	23	22	24
Direct TLC Bioautography results	Retardation factors (R*_f_*) of inhibition zones.	0.20, 0.40, 0.70	0.30, 0.40, 0.55, 0.70, 0.80	0.40, 0.55, 0.70	0.30
Color indicator Legend	0–5 mm	5–10 mm	10–15 mm	15–20 mm	>20 mm

^a–d^ means with same superscript letters are not significantly different (*p* > 0.05) within the same column for a particular fraction; ^e^ Values represent mean and standard deviation (*n* = 3).

**Table 3:** Ferric reducing antioxidant power of different fractions.

Concentration (µg/mL)	Standard (%)	Fraction I (%)	Fraction II (%)	Fraction III (%)	Fraction IV (%)	Fraction V (%)
10	50.3 ± 0.1 ^c^	31.1 ± 0.9 ^a^	30.1 ± 0.8 ^a^	52.9 ± 0.6 ^b^	37.0 ± 0.6 ^b^	42.7 ± 0.3 ^b^
20	71.7 ± 1.3 ^e^	39.7 ± 0.4 ^b^	37.6 ± 0.9 ^b^	70.5 ± 0.4 ^e^	49.9 ± 0.1 ^c^	61.9 ± 0.5 ^d^
40	86.7 ± 1.3 ^g^	49.8 ± 0.5 ^c^	41.6 ± 0.7 ^b^	89.1 ± 0.6 ^g^	70.1 ± 0.3 ^e^	81.0 ± 0.5 ^f^
80	92.9 ± 0.5 ^h^	69.5 ± 0.6 ^e^	50.7 ± 0.4 ^c^	95.8 ± 0.2 ^h^	80.0 ± 0.4 ^f^	89.0 ± 0.6 ^g^
160	98.5 ± 0.5 ^i^	80.6 ± 0.3 ^f^	67.1 ± 0.3 ^e^	98.8 ± 0.2 ^i^	86.9 ± 0.1 ^g^	93.1 ± 0.3 ^h^

^a–i^ means with same superscripts are not significantly different (*p* > 0.05).

**Table 4:** Standards of phenolic compounds and their retention time.

Standards	Retention Time (min)	Concentration (ppm)	Area ^1^
**Hydroquinone**	2.53	25	128,153,656 (0.01)
**4-** **Hydroxy benzoic acid**	2.85	25	43,264,890 (0.01)
**Vanillic acid**	3.07	25	26,209,327 (0.01)
**Catechol**	2.78	25	72,980,280 (0.02)
**Quercetin**	3.26	25	30,545,891 (0.01)
**Gallic acid**	2.06	25	25,719,999 (0.01)
**Cinnamic acid**	3.62	25	5,012,145 (0.01)
**Thymol**	3.32	25	8,398,173 (0.02)

^1^ Area represented as mean (*n* = 5) with coefficient of variation in brackets.

## Data Availability

Data is contained within the article
